# Multiple Introduction and Naturally Occuring Drug Resistance of HCV among HIV-Infected Intravenous Drug Users in Yunnan: An Origin of China’s HIV/HCV Epidemics

**DOI:** 10.1371/journal.pone.0142543

**Published:** 2015-11-12

**Authors:** Min Chen, Yanling Ma, Huichao Chen, Hongbing Luo, Jie Dai, Lijun Song, Chaojun Yang, Jingyuan Mei, Li Yang, Lijuan Dong, Manhong Jia, Lin Lu

**Affiliations:** 1 Institute for AIDS/STD Control and Prevention, Yunnan Center for Disease Control and Prevention, Kunming, Yunnan, 650022, China; 2 College of Public Health, Kunming Medical University, Kunming, Yunnan, 650500, China; Fudan University, CHINA

## Abstract

**Background:**

The human immunodeficiency virus 1 (HIV-1) epidemic in China historically stemmed from intravenous drug users (IDUs) in Yunnan. Due to a shared transmission route, hepatitis C virus (HCV)/HIV-1 co-infection is common. Here, we investigated HCV genetic characteristics and baseline drug resistance among HIV-infected IDUs in Yunnan.

**Methods:**

Blood samples of 432 HIV-1/HCV co-infected IDUs were collected from January to June 2014 in six prefectures of Yunnan Province. Partial *E1E2* and *NS5B* genes were sequenced. Phylogenetic, evolutionary and genotypic drug resistance analyses were performed.

**Results:**

Among the 293 specimens successfully genotyped, seven subtypes were identified, including subtypes 3b (37.9%, 111/293), 3a (21.8%, 64/293), 6n (14.0%, 41/293), 1b (10.6%, 31/293), 1a (8.2%, 24/293), 6a (5.1%, 15/293) and 6u (2.4%, 7/293). The distribution of HCV subtypes was mostly related to geographic location. Subtypes 3b, 3a, and 6n were detected in all six prefectures, however, the other four subtypes were detected only in parts of the six prefectures. Phylogeographic analyses indicated that 6n, 1a and 6u originated in the western prefecture (Dehong) and spread eastward and showed genetic relatedness with those detected in Burmese. However, 6a originated in the southeast prefectures (Honghe and Wenshan) bordering Vietnam and was transmitted westward. These subtypes exhibited different evolutionary rates (between 4.35×10^−4^ and 2.38×10^−3^ substitutions site^-1^ year^-1^) and times of most recent common ancestor (tMRCA, between 1790.3 and 1994.6), suggesting that HCV was multiply introduced into Yunnan. Naturally occurring resistance-associated mutations (C316N, A421V, C445F, I482L, V494A, and V499A) to NS5B polymerase inhibitors were detected in direct-acting antivirals (DAAs)-naïve IDUs.

**Conclusion:**

This work reveals the temporal-spatial distribution of HCV subtypes and baseline HCV drug resistance among HIV-infected IDUs in Yunnan. The findings enhance our understanding of the characteristics and evolution of HCV in IDUs and are valuable for developing HCV prevention and management strategies for this population.

## Introduction

Hepatitis C virus (HCV) is a single-stranded positive RNA virus that has been categorized into the Hepacivirus genus of the Flaviviridae family. Persistent HCV infection is associated with the development of liver cirrhosis, hepatocellular carcinoma, liver failure, and death [1]. About 3% of the world population is infected with HCV, with a total of about 170 million carriers. While HCV infection is found worldwide, Central and East Asia and North Africa/Middle East are the most affected regions, where the HCV seroprevalence is >3.5% [2]. In China, the prevalence of anti-HCV antibodies was estimated to be 3.2% in the Chinese population, equating to 40 million infections [3].

Based on phylogenetic and sequence analyses of whole viral genomes, HCV strains are classified into seven recognized genotypes. Except for genotypes 5 and 7, each genotype is further divided into a variable number of subtypes, including 67 confirmed and 20 provisional subtypes [4]. Globally, genotype 1 is the most prevalent, followed by genotype 3, 2, 4 and 6. Genotype 5 and 7 are the least prevalent [5, 6]. Different genotypes/subtypes have distinct geographic distribution patterns. Subtypes 1a, 1b, 2a and 3a are widely distributed across the globe [7]. In contrast, other HCV subtypes that have circulated in restricted regions are known as endemic subtypes. In general, endemic subtypes from genotypes 1 and 2 are primarily found in West Africa, 3 is in South Asia, 4 is in Central Africa and the Middle East, 5 is in Southern Africa, and 6 is in South East Asia [8]. In China, over 95% of the isolates belong to five major subtypes: 1b, 2a, 6a, 3a and 3b. Among them, subtype 1b is the most prominent nationwide, which is followed by 2a [9, 10]. A new trend is the increased prevalence of 6a in south China [11, 12].

Understanding HCV genetic variation is important in a number of areas. First, HIV variation has epidemiological implications. In general, the distribution of HCV genotypes/subtypes is associated with transmission routes, human migrations and specific public health events [2, 5, 6, 13]. Additionally, HCV genotypes have important clinical implications. In practice, the duration of treatment, cure rates, and the need for adjuvant interferon and ribavirin depend on HCV genotype/subtype. Furthermore, the genetic diversity of HCV present a challenge for the development of an HCV vaccine. The selection and design of vaccine immunogens require a comprehensive understanding of the prevalence of region-specific HCV subtypes.

As a bloodborne virus, HCV is most commonly transmitted through injection drug use, blood transfusions and sexual contact [14]. Due to shared transmission routes, co-infection with HCV and HIV-1 has become common among individuals who have a high risk of blood exposure. Especially, intravenous drug users (IDUs) is the most important route for co-infection. Among HIV-infected IDUs, the HCV seroprevalence rate can be as high as 90% [15]. Whether HIV-1/HCV co-infection results in a faster progression to AIDS remains controversial [16], however, co-infection can accelerate the progression of HCV-related liver disease [15, 17, 18]. Furthermore, with the application of highly active antiretroviral therapy (HAART), HIV-related complications have been suppressed among patients with co-infection, causing the impact of HCV to become more apparent. HCV is the most common cause of death among HIV-positive patients treated with HAART [18]. To decrease the mortality and morbidity of HCV among HIV chronically infected IDUs, the treatment for hepatitis C is necessary.

Recently, the rapid development of direct-acting antivirals (DAAs) is revolutionizing HCV pharmaceutical treatment [19, 20]. DAAs target specific nonstructural proteins of HCV and results in disruption of viral replication and infection. According to their action sites, DAAs are classed as NS3/4A protease inhibitors (PIs), NS5B nucleoside polymerase inhibitors (NIs), NS5B non-nucleoside polymerase inhibitors (NNIs), and NS5A inhibitors [21]. Comparing with the previous pegylated interferon-α (PegIFN-α) and ribavirin (RBV)-based regimens, the DAA regimens significantly improved the rate of sustained virologic response for most populations, including several unique patient populations, such as patients with HIV/HCV dual infection [22]. Importantly, the DAA regimens decreased patient co-morbidities and adverse events associated with IFN-based therapy in the HIV/HCV-infected population. However, the mutations resistant to DAAs arose rapidly during therapy in early clinical trials [23]. Furthermore, naturally occuring resistance-associated mutations to HCV protease and polymerase inhibitors could be found in treatment-naïve patients [24–26]. Thus, drug resistance is still a challenge for the treatment of HCV infection.

Yunnan province is located in southwest China and is situated along drug trafficking routes channeling heroin into China. Since the identification of the first HIV epidemic in China among intravenous drug users (IDUs) in Yunnan in 1989, Yunnan has been one of the areas hardest hit by HIV in China [27]. In the early stages, intravenous drug use was the predominant transmission route, and had driven the HIV epidemic in Yunnan. According to data obtained by sentinel surveillance in Yunnan, the HIV and HCV seroprevalence rates among IDUs were 19.54% and 53.56%, respectively. In addition, 16.08% of IDUs were reported as co-infected with HIV and HCV.

In the present study, we performed a cross-sectional HCV molecular epidemiological and baseline drug resistance investigation among IDUs co-infected with HCV and HIV-1 in Yunnan Province. Seven subtypes of three genotypes were detected, which showed unique geographic distributions and genetic characteristics. Results suggest that HCV was multiply introduced into Yunnan by IDUs at different times. Naturally occuring resistance-associated mutations to HCV NS5B polymerase inhibitors were detected in selected subtypes. These findings provide new insights into HCV transmission and information for HCV management and treatment in Yunnan.

## Materials and Methods

### Study participants and sample collection

A total of 432 blood samples of IDUs positive for anti-HCV and anti-HIV-1 antibodies were continuously collected between January and June 2014 through fixed sentinel surveillance sites in 6 prefectures of Yunnan Province, including Dehong, Lincang, Dali, Kunming, Honghe and Wenshan, where reported HIV-1 cases account for 70% of total cases in Yunnan. HIV-1 infection status was determined by an Enzyme-Linked Immunosorbent Assay (ELISA, Biomerieux, France) and confirmed by Western blot assay (HIV BLOT 2.2, MP Diagnostics, Singapore). Anti-HCV was screened and re-tested with two different ELISA kits (Intec (Xiamen) Technology Co., Ltd., Fujian, China and Beijing Wantai Biological Pharmacy Enterprise Co., Ltd., Beijing, China). Participation was voluntary and written consents were obtained from all participants. The study was approved by Biomedical Ethics Review Committee of Yunnan Center for Disease Control and Prevention.

### Amplification of HCV gene fragments

Plasma was separated from whole blood. Viral RNA was extracted from 140 μl of plasma by using the QIAamp Viral RNA Mini kit (Qiagen, Valencia, CA, United States) according to the manufacturer’s instructions, and was then subjected to nested polymerase chain reaction (PCR) to amplify the fragments of the *E1E2* (H77: 933–2060) and *NS5B* (H77: 8340–9233). The primers and conditions of PCR reaction were described previously [28]. The first PCR reaction was performed using One Step reverse transcription PCR (Takara, Dalian, China). The second PCR reaction was performed using 2×Taq PCR MasterMix (Tiangen, Beijing, China). PCR products were sent to ZIXIBIO Co. (Beijing, China) for sequencing using an ABI 3730XL automated DNA sequencer (Applied Biosystems, Carlsbad, USA).

### Sequence analysis

The original sequences were assembled with DNA sequence analysis software Sequencher 5.0 (Gene Codes, Ann Arbor, MI). The ClustalW Multiple alignment and manual editing were performed using Bio-Edit 7.0 software. The reference sequences were obtained from the HCV database located in Los Alamos (http://hcv.lanl.gov/content/sequence/HCV/ToolsOutline.html). Some sequences of IDUs from Yunnan, Jiangsu and Henan were included [28–30]. Phylogenetic tree analyses were performed using the neighbor-joining method based on Kimura two-parameter model with 1000 bootstrap replicates, using MEGA (Molecular Evolutionary Genetics Analysis, version 5.1) [31]. Genetic distances of pair sequences within each subtype are calculated on E1E2 and NS5B fragments using the Kimura two-parameter model. The analysis of NS5B resistance-associated mutations was described previously [24]. A total of 18 positions related to drug resistance to NS5B polymerase inhibitors were analyzed at the amino acid sequence level. The HCV 1b reference sequence (GenBank accession number AJ238799) was used for the definition of amino acid substitutions.

### Geographic distribution analysis of HCV subtypes

HCV genetic geographic distribution was analyzed with the public health geographic information system (PHGIS, China CDC). A dot density map was used to display the distribution density of each subtype within each prefecture. For each subtype, the number of patients with the subtype in one prefecture was divided by the total number of patients with definite subtype in this prefecture to obtain the constituent ratio of each subtype in each prefecture. When using PHGIS to map the data, one dot was defined as 0.025%.

### Bayesian MCMC evolutionary analyses

The evolution rate and time of most recent common ancestor (tMRCA) for each HCV subtype among IDUs in Yunnan were inferred from the *NS5B* gene using Bayesian Markov chain Monte Carlo (MCMC) method. The general time reversible (GTR) model plus a gamma distribution (Γ4) among site rate heterogeneity (I) model (GTR+I+Γ4) was evaluated as the best nucleotide substitution model for all datasets by the jModeltest version 2.1.2. Bayesian MCMC analyses were performed using a Bayesian uncorrelated exponential relaxed molecular clock method in combination with four different coalescent tree priors (‘Constant Size’; ‘Exponential Growth’, ‘Logistic Growth’ and ‘Bayesian Skyline’) under the selected nucleotide substitution model in the BEAST v1.7.4 package [32]. Each MCMC analysis was run for 20 million generations and sampled every 2,000 generations. The resulting log-files were analyzed in Tracer v1.5 and the Bayes Factor was calculated to compare molecular clock models, using marginal likelihood as implemented in Tracer v.1.5 (S1 Table). Based on Bayes Factors, the uncorrelated exponential relaxed molecular clock model with Bayesian Skyline coalescent tree prior was the best-fitting model for subtype 1b, 3a, 3b, 6a, 6n and 6u, the uncorrelated exponential relaxed molecular clock model with Exponential Growth coalescent tree prior was the best-fitting model for subtype 1a. The MCMC analyses with the best-fitting model for selected subtype were re-run for 50 million generations and sampled every 5,000 generations. The Maximum Clade Credibility (MCC) tree was obtained by TreeAnnotator v1.7.4 with a burn-in of the initial 10% of generated trees, and examined by FigTree V1.3.1, which was also used to estimate the evolutionary rates and the dates to tMRCA of various nodes on the MCC tree.

### Sequence data

All the sequences obtained in this study were submitted to GenBank under accession numbers KT735394 to KT735898.

### Statistical analysis

Statistical analyses were conducted using the SPSS 19.0 statistical analysis software package (SPSS Inc. Chicago, IL). Categorical variables were compared using χ^2^ tests. All tests were two-tailed and a *p*-value <0.05 was considered statistically significant.

## Results

### Demographic characteristics of study subjects

**Fig 1:**
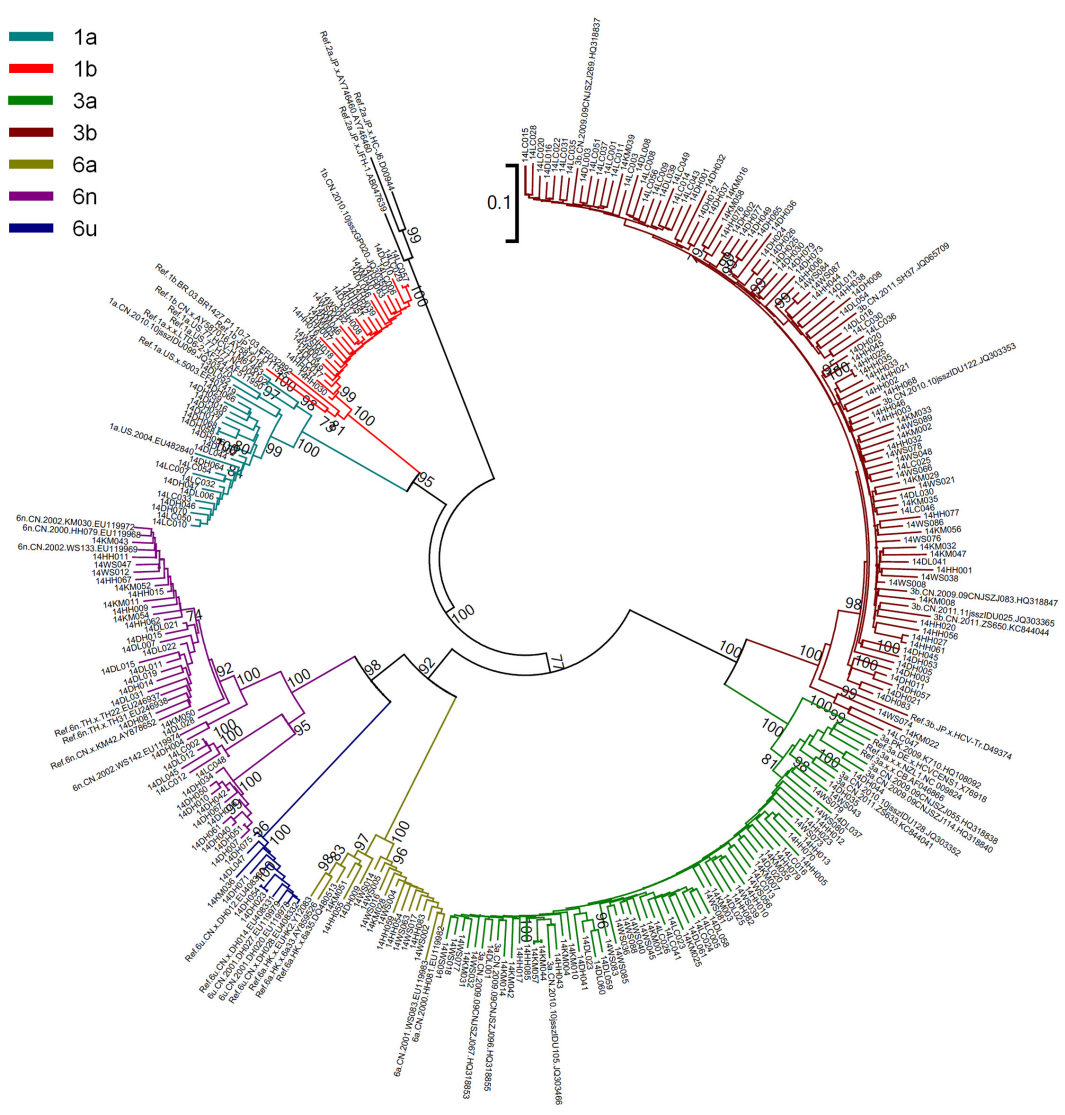
Neighbor-joining phylogenetic tree of partial *E1E2* gene from HIV/HCV co-infected IUDs in Yunnan. Neighbor-joining phylogenetic tree for 265 *E1E2* sequences and relative reference sequences. The scale bar indicates 10% nucleotide sequence divergence. Values on the branches represent the percentage of 1000 bootstrap replicates and bootstrap values over 70% are shown in the tree.

**Fig 2:**
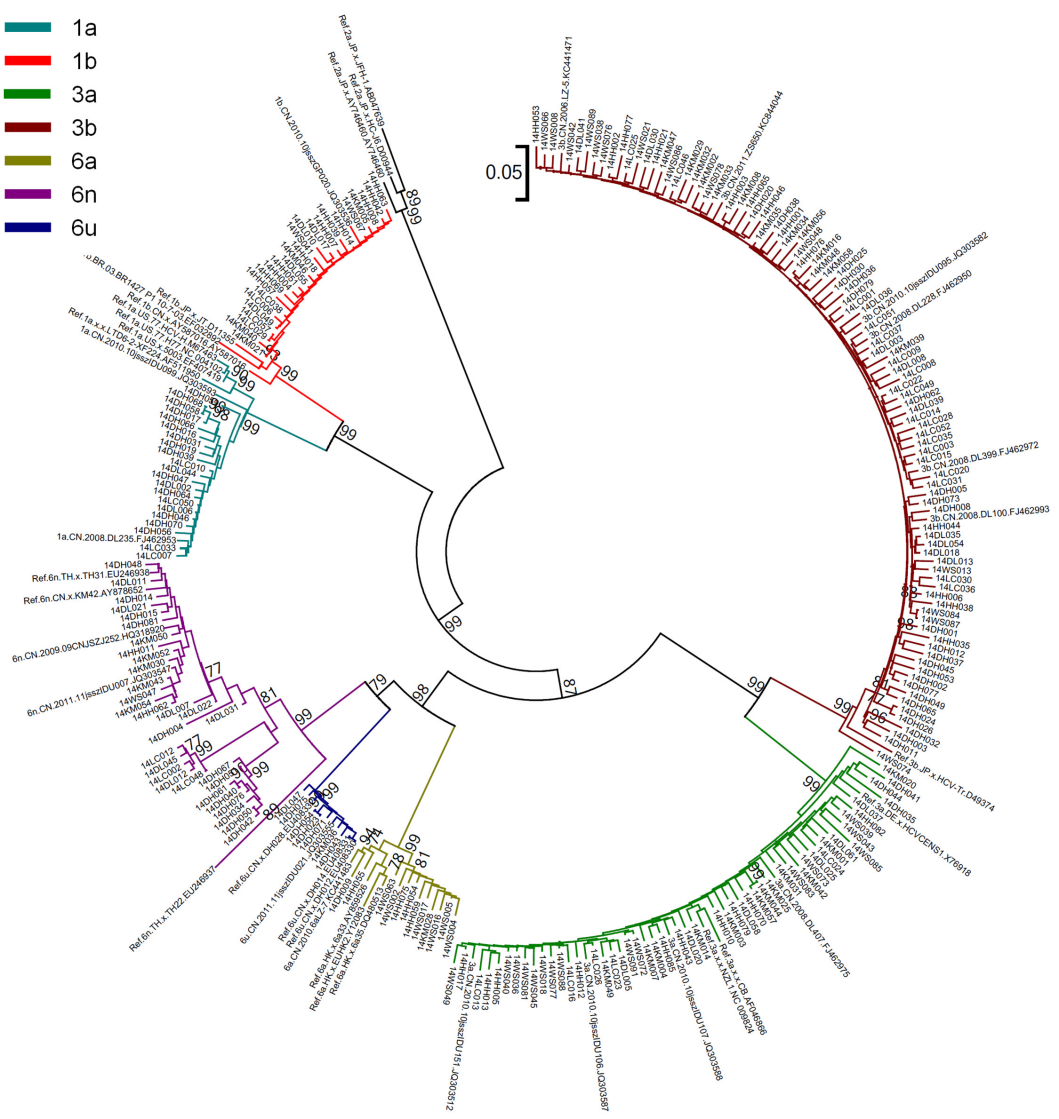
Neighbor-joining phylogenetic tree of partial *NS5B* gene from HIV/HCV coinfected IUDs in Yunnan. Neighbor-joining phylogenetic tree for 240 *NS5B* sequences and relative reference sequences. The scale bar indicates 5% nucleotide sequence divergence. Values on the branches represent the percentage of 1000 bootstrap replicates and bootstrap values over 70% are shown in the tree.

A total of 432 HCV seropositive blood samples were collected from IDUs infected with HIV-1 in 6 prefectures of Yunnan Province, including Dehong (82), Lincang (57), Dali (61), Kunming (56), Honghe (85) and Wenshan (91). All of these samples were subjected to HCV genotyping. For each sample, the viral *E1E2* and *NS5B* genes were amplified and sequenced. In total, 265 *E1E2* sequences and 240 *NS5B* sequences were obtained. By combining the phylogenetic tree analyses of *E1E2* and *NS5B* (Figs 1 and 2), 293 samples were successfully genotyped.

Among the 293 subjects with data that was successfully genotyped, the ratio of males to females was 1:0.14. The mean age was 37.4 years (range: 16–58 years); And 63.8% (187/293) of individuals were of Han ethnicity, and 36.2% of individuals were minority nationality, including Dai, Jingpo, Hui, Yi, Zhuang, Bai, Deang, Menggu, Wa and Lagu. Of them, 45.1% (132/293) were single, 34.8% (102/293) were married, 17.4% (51/293) were divorced or widowed, and 2.7% (8/293) did not disclose their marital status. Of the 293 subjects, 84.0% (246/293) were Chinese, and 16.0% (47/293) were Burmese who stayed in Dehong Prefecture.

### Prevalent HCV subtypes among IDUs infected with HIV

Three HCV genotypes (1, 3 and 6) were identified, which included seven HCV subtypes (1a, 1b, 3a, 3b, 6a, 6n and 6u). Among IDUs in Yunnan, HCV genotype 3 (59.7%, 175/293) was the most predominant, followed by genotype 6 (21.5%, 63/293) and genotype 1 (18.8%, 55/293). For all the subtypes detected in this study, subtype 3b (37.9%, 111/293) was the most common; the other subtypes included 3a (21.8%, 64/293), 6n (14.0%, 41/293), 1b (10.6%, 31/293), 1a (8.2%, 24/293), 6a (5.1%, 15/293) and 6u (2.4%, 7/293).

### Distribution characteristic of HCV subtypes among IDUs in Yunnan

The demographic study revealed that the distribution of HCV subtypes by the participants’ gender, age, and marital status showed no statistical differences (Table 1). However, the distribution of subtypes by geographical location (the prefectures where the samples were collected), ethnicity and nationality showed a statistical difference. Furthermore, the distributions of the participants’ ethnicity and nationality were dependent upon their resident location (Table 2). Thus, the distribution of HCV subtypes was mostly related to geographical location.

**Table 1:** Demographic characteristics and HCV subtypes of study subjects.

		Subjects	Subtypes							χ2	*P*
			1a	1b	3a	3b	6a	6n	6u		
Total		293	24	31	64	111	15	41	7		
Prefecture										<0.001	<0.001
	Dehong	65	14	0	3	27	1	15	5		
	Lincang	44	6	5	7	23	0	3	0		
	Dali	41	4	5	10	11	0	10	1		
	Kunming	41	0	4	14	14	2	6	1		
	Honghe	57	0	14	11	22	5	5	0		
	Wenshan	45	0	3	19	14	7	2	0		
Gender										6.335	0.345
	Male	258	23	28	53	101	12	34	7		
	Female	35	1	3	11	10	3	7	0		
Age										7.907	0.242
	≤35	155	18	18	29	57	6	23	4		
	≥36	138	6	13	35	54	9	18	3		
Marital Status										11.072	0.523^a^
	Unmarried	132	11	15	24	54	9	16	3		
	Married	102	9	11	29	31	2	16	4		
	Divoiced/Widowed	51	4	5	10	21	4	7	0		
	Unknown	8	0	0	1	5	0	2	0		
Race/Ethnicity										22.678	0.001
	Han	187	12	23	49	73	8	22	0		
	Others	106	12	8	15	38	7	19	7		
Nationality										51.783	<0.001
	Chinese	246	11	31	63	92	15	31	3		
	Burmese	47	13	0	1	19	0	10	4		

a Compared the constitutions of subtypes among Unmarried, Married and Divoiced/Widowed.

**Table 2:** The distributions of the participants’ ethnicity and nationality by their resident location.

		Subjects	Prefectures						χ2	*P*
			Dehong	Lincang	Dali	Kunming	Honghe	Wenshan		
Total		293	65	44	41	41	57	45		
Race/Ethnicity									81.551	<0.001
	Han	187	15	38	32	38	42	22		
	Others	106	50	6	9	3	15	23		
Nationality									196.359	<0.001
	Chinese	246	18	44	41	41	57	45		
	Burmese	47	47	0	0	0	0	0		

**Fig 3:**
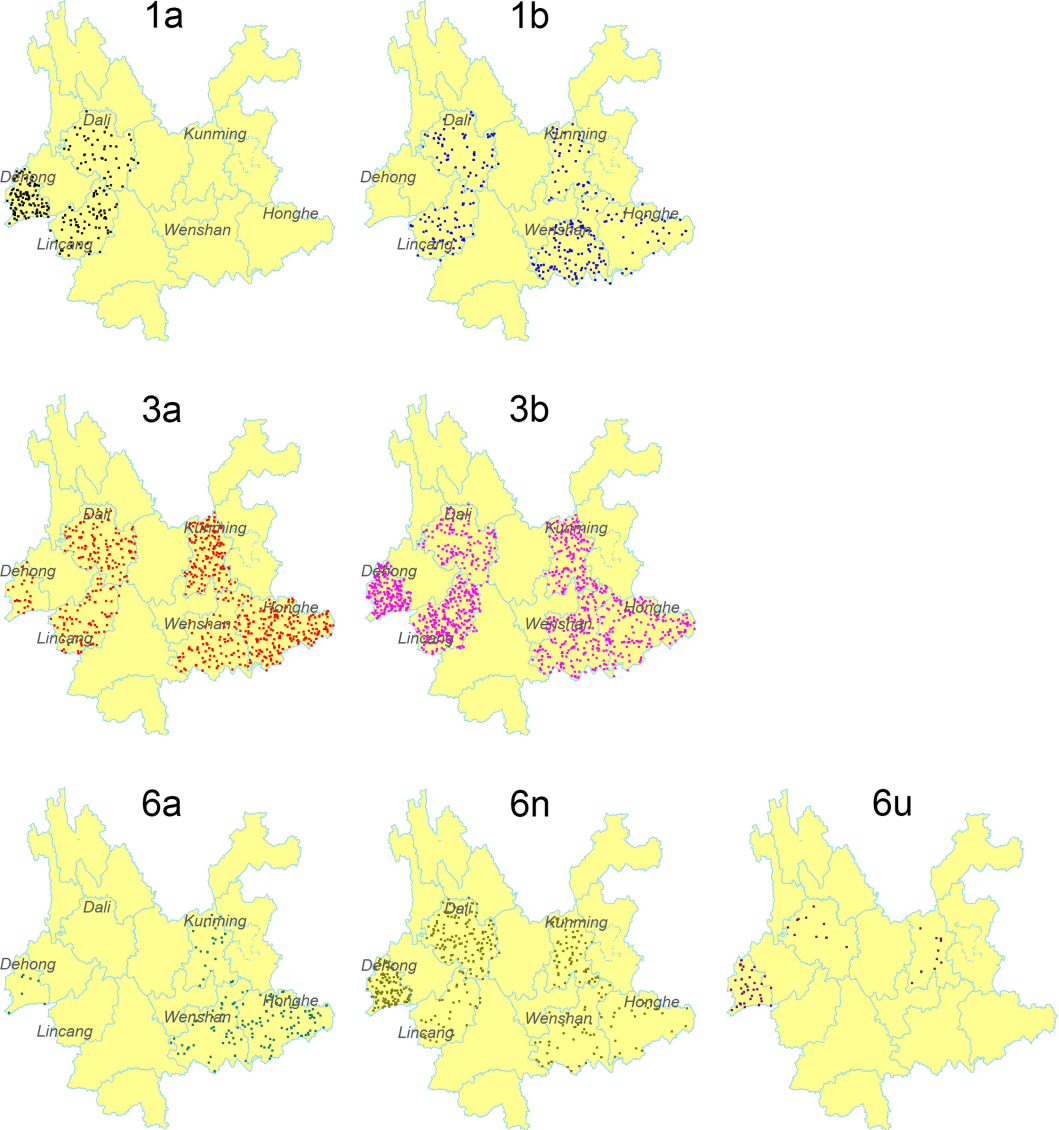
Geographic distribution of the HCV subtypes in Yunnan. Dot Density Map for 1a, 1b, 3a, 3b, 6a, 6n and 6u, respectively, which showed the constituent ratio of each subtype in each prefecture. One dot represents 0.025% of the subjects in each prefecture.

As shown in Fig 3, subtype 3b, 3a and 6n were found among the IDUs in all six prefectures. Subtype 3b was the predominant subtype in each prefecture. The constituent ratio of subtype 3a showed a decreasing trend from the eastern prefectures to the western ones, however, the changing trend of subtype 6n was the opposite of this. Subtype 1b was detected in five prefectures except Dehong. In the early 2000s, subtype 1a and 6u were just found among IUDs in Dehong [29], a western prefecture bordering with Myanmar. In this study, it was found that these two subtypes spread to the internal area of Yunnan Province (Dali and Kunming). Traditionally, subtype 6a was limited among IDUs in Honghe and Wenshan, two prefectures bordering with Vietnam. In this study, in addition to Honghe and Wenshan, separate IDUs with subtype 6a were found in Kunming and Dehong.

**Fig 4:**
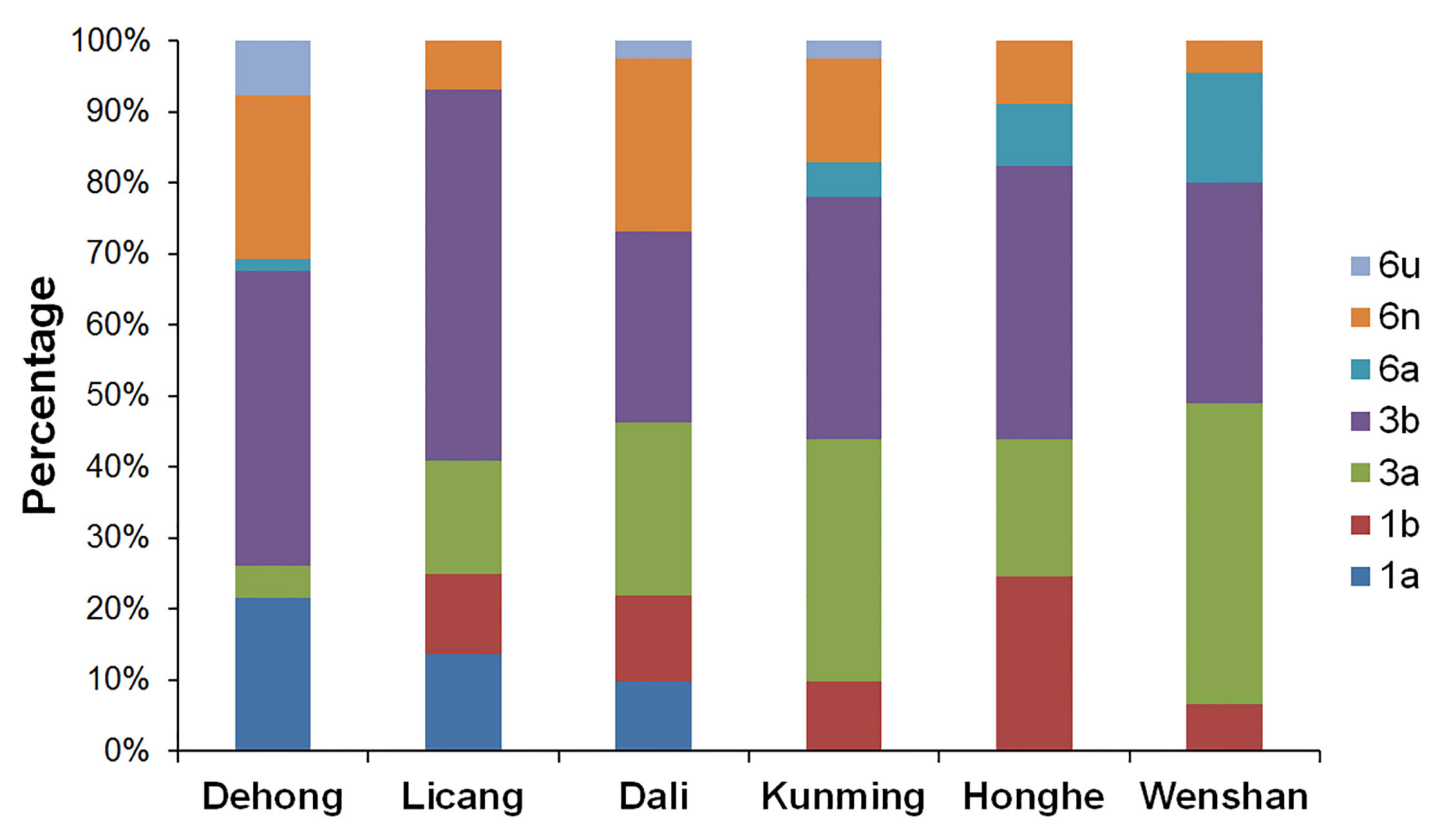
The Distribution of HCV subtypes for six prefectures in Yunnan. The constitution of HCV subtypes for each prefecture was showed.

For each prefecture, the constitution of HCV subtypes was complex (Fig 4). Five HCV subtypes were found in Lincang, Honghe and Wenshan, and six HCV subtypes in Dehong, Dali and Kunming.

### HCV genetic characteristics among IDUs in Yunnan

Pairwise genetic distances within each subtype were calculated with *E1E2* and *NS5B* sequences using the Kimura two-parameter model (Table 3). For both *E1E2* and *NS5B* sequences, the mean genetic distances within subtype 6n were the largest. The mean genetic distances within subtype 3b, 3a and 6a were medial. However, the mean genetic distances within subtype 1b, 6u and 1a were smaller than those within the other four subtypes.

**Table 3:** Genetic Distances among Sequences belonging to Different HCV subtypes.

	Genetic Distances (mean ± SE)						
	1a	1b	3a	3b	6a	6n	6u
*E1E2*	0.0710±0.0021# & $ ^	0.0540±0.0009* & $ ^	0.0911±0.0005* # $ ^	0.1142±0.0005* # & ^ %	0.0874±0.0042* # $	0.1958±0.0039* # & $ ^ %	0.0639±0.0063$
*NS5B*	0.0267±0.0007# & $ ^	0.0184±0.0005* & $ ^	0.0376±0.0004* # $ ^ %	0.0421±0.0002* # & %	0.0454±0.0030* # & %	0.0817±0.0020* # & $ ^ %	0.0194±0.0013& $ ^

*: p<0.05, when comparing with the counterpart of 1a

#: p<0.05, when comparing with the counterpart of 1b

&: p<0.05, when comparing with the counterpart of 3a

$: p<0.05, when comparing with the counterpart of 3b

^: p<0.05, when comparing with the counterpart of 6a

%: p<0.05, when comparing with the counterpart of 6u

To estimate the evolutionary rates and the tMRCA of HCV subtypes, MCMC analyses were performed using *NS5B* sequences from this study and previous work [29]. Although the evolutionary rates in *NS5B* region were low, there were still differences among different subtypes (Table 4). The median evolutionary rates of these HCV subtypes were between 4.35×10^−4^ and 2.38×10^−3^ substitutions site^-1^ year^-1^. The evolutionary rate of 1a was the highest, and that of 6n was the lowest. The median tMRCA for each subtype are shown in Table 4. The median tMRCA of subtype 6n circulating in Yunnan was 223.7 years ago (1790.3), the earliest among these subtypes. However, the latest median tMRCA was for subtype 6u, which was 19.4 years ago (1994.6).

**Table 4:** The evolutionary rates and the times of most recent common ancestor (tMRCA) of HCV subytpes.

		1a	1b	3a	3b	6a	6n	6u
tMRCA								
	median	1991.7	1991.4	1973.4	1944.1	1939.9	1790.3	1994.6
	95% HPD lower	1999.4	1996.3	1991.7	1994.6	1997.9	1974.0	2000.3
	95% HPD upper	1980.5	1977.8	1917.7	1748.9	1552.0	481.6	1972.3
meanRate								
	median	2.38×10^−3^	8.78×10^−4^	1.54×10^−3^	8.16×10^−4^	7.26×10^−4^	4.35×10^−4^	2.31×10^−3^
	95% HPD lower	1.21×10^−4^	3.47×10^−6^	2.27×10^−6^	5.45×10^−7^	1.59×10^−7^	1.81×10^−8^	4.99×10^−6^
	95% HPD upper	5.36×10^−3^	2.08×10^−3^	5.13×10^−3^	3.90×10^−3^	4.15×10^−3^	2.49×10^−3^	9.15×10^−3^

HPD: the highest posterior density

### Phylogeographic tree analysis

The maximum clade credibility (MCC) trees were reconstructed with the trees sampled during MCMC analyses. The MCC tree of subtype 3b, 3a and 1b presented a substantial mixture of geographic origins (data not shown), indicating that they have widely circulated in Yunnan and have no apparent geographical trend in transmission.

**Fig 5:**
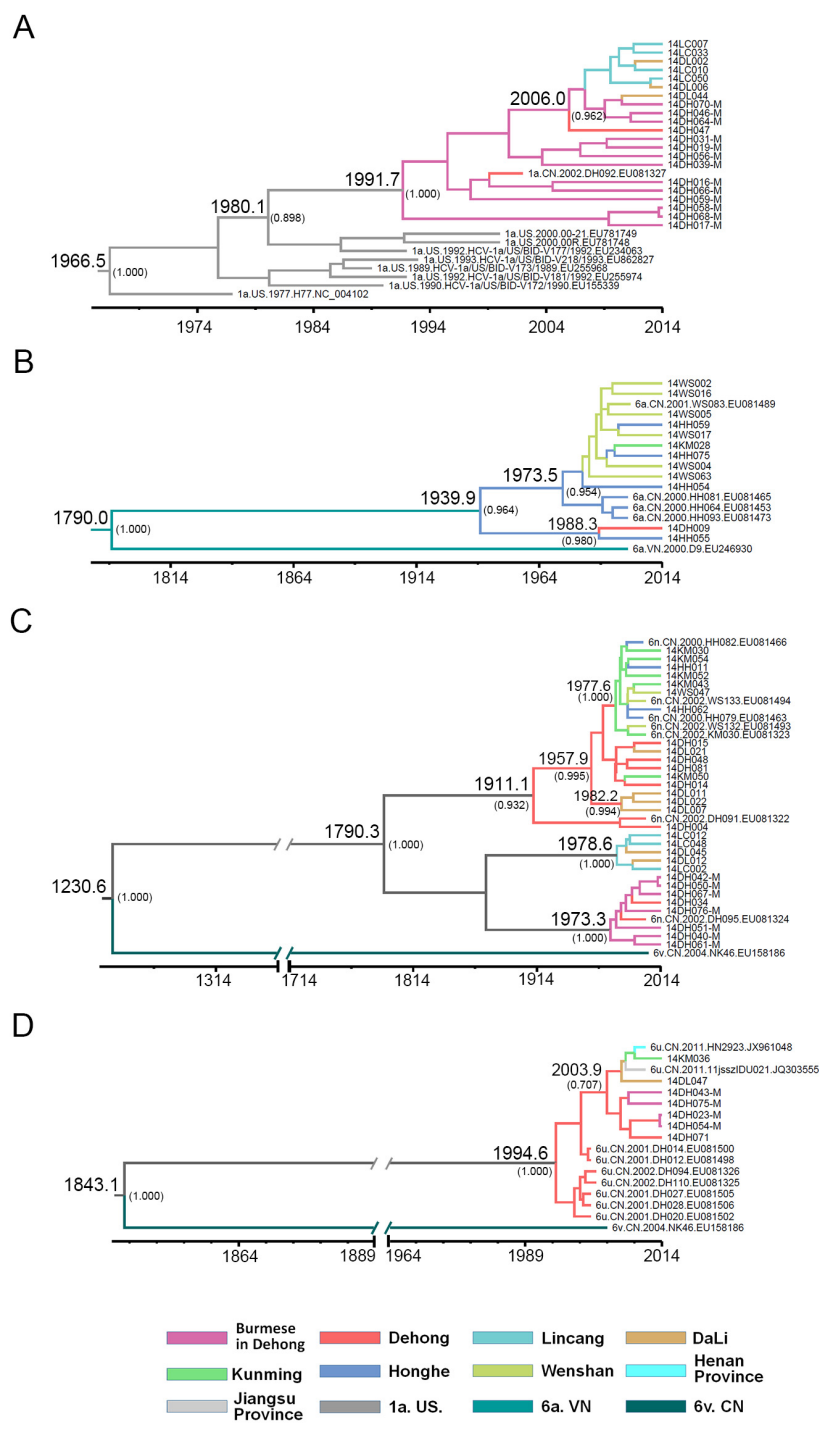
Maximum clade credibility (MCC) tree representing the rooted genealogy of HCV sutypes 1a, 6a, 6n and 6u among HIV/HCV coinfected IDUs in Yunnan. (A) The MCC tree for subtype 1a strains. (B) The MCC tree for subtype 6a strains. (C) The MCC tree for subtype 6n strains. (D) The MCC tree for subtype 6u strains. The MCC trees were obtained by Bayesian MCMC analysis based on partial *NS5B* gene (H77: 8340–9233) implemented in BEAST v 1.7.4. The uncorrelated exponential relaxed molecular clock method was used in combination with the Bayesian Skyline coalescent tree prior under GTR+I+G4 nucleotide substitution model. The branch lengths in the MCC trees reflect time and the corresponding time-scale is shown at the bottom of the trees. The posterior probabilities of the key nodes and the tMRCA medians for the interested nodes are indicated.

MCC tree of subtype 1a showed that the isolates from Burmese in Dehong were the direct descendants of the most recent 1a common ancestor dated around 1991 (Fig 5A). Branched from the clades of Burmese, a cluster including the isolates from Burmese in Dehong and Chinese in Dehong, Lincang and Dali descended from a common ancestor dated around 2006. These suggested that 1a originated from Myanmar, and transmitted among IDUs from Dehong to Lincang and Dali.

In the MCC tree of subtype 6a (Fig 5B), the isolates from Yunnan and Vietnam shared a common ancestor dated around 1790. The isolates from Yunnan formed two clusters, which descended from a most recent common ancestor dated around 1939. The tMRCA of one cluster was 1973, which mainly included the isolates from Honghe and Wenshan, two prefectures bordering with Vietnam. Further, one isolate from Kunming seemed to derive from the isolate circulating in Honghe. In the other cluster, one isolate from Dehong clustered with high posterior probability with a isolate from Honghe, whose tMRCA was 1998, which could be a long-distance transmission.

The MCC tree of subtype 6n included three independent clusters with high posterior probabilities (Fig 5C). The three clusters descended from a most recent common ancestor dated around 1790. The tMRCA of Cluster I was 1911, in which two isolates from Dehong were positioned at the base, and were the most direct descendents. Further, a common ancestor dated around 1957 from Dehong began transmitting to Dali, Kunming, Honghe and Wenshan. These suggested that 6n in Cluster I origined in Dehong, and spread to the other prefectures. Cluster II and III were younger than Cluster I, whose tMRCA were around 1978 and 1973, respectively. In Cluster II, 6n was first introduced into IDUs of Lincang, and then into IDUs of Dali. However, the isolates in Cluster III were limited in Dehong, most of which were from Burmese in Dehong. All of these findings suggest that 6n has circulated for a long time in the China- Myanmar bordering area and were multiply introduced.

In the MCC tree of 6u (Fig 5D), all isolates descended from a common ancestor dated around 1994, most of which were from Dehong. Branched from the isolated from Dehong, a cluster descended from a common ancestor dated around 2003, which included the isolates from Dali, Kunming and the other provinces (Henan and Jiangsu). These findings suggest that 6n initiated in Dehong and was transmitted to the other prefectures and even to the other provinces.

### Resistance-associated mutations to inhibitors of NS5B ploymerase

The available 240 *NS5B* sequences were screened for resistance-associated mutations in DAAs treatment-naïve IDUs co-infected with HCV and HIV. Variation in amino acid composition was studied at 18 positions known to be associated with resistance to polymerase inhibitors (Table 5). Among the 18 NI/NNI resistance-associated positions, 12 were not detected resistance-associated variations (RAVs) among all the subtypes found in this study, including S282, P495, P496, L419, R422, M423, M426, A486, S368, M414, Y448 and Y452. However, at the other 6 positions, in spite of being minor variants, naturally occuring RAVs were found with high prevalence in some subtypes: 421V in 100% of 3a, 6a, 6n and 6u, 97.8% of 3b and 4.0% of 1b; 449A in 100% of 1a, 3a, 3b, 6a, 6n and 6u, and 8.0% of 1b; 482L in 100% of 3a, 3b, 6a, 6n and 6u; 494A in 100% of 6n and 6u, 8.3% of 6a, and 1.1% of 3b; 316N in 100% of 1b; 445F in 100% of 3a, 6a, 6n and 6u, 98.9% of 3b, 4.8% of 1a and 4.0% of 1b.

**Table 5:** Resistance-associated amino acid subsititutions to inhibitors of NS5B polymerase.

Subtype	Subjects	NI	NNI-1				NNI-2							NNI-3/4					
		S282T	A421V	P495L/S/A/T/Q	P496S	V499A	L419S	R422K	M423T/V/I	M426A/T	I482S/L	A486V	V494A	C316Y/N	S368T	M414T/L	C445F	Y448H/C	Y452H
1a	21	0	0	0	0	21(100.0%)	0	0	0	0	0	0	0	0	0	0	1(4.8%)	0	0
1b	25	0	1(4.0%)	0	0	2(8.0%)	0	0	0	0	0	0	0	25(100.0%)^1^	0	0	1(4.0%)	0	0
3a	51	0	51(100.0%)	0	0	51(100.0%)	0	0	0	0	51(100.0%)^2^	0	0	0	0	0	51(100.0%)	0	0
3b	93	0	91(97.8%)	0	0	93(100.0%)	0	0	0	0	93(100.0%)^2^	0	1(1.1%)	0	0	0	92(98.9%)	0	0
6a	12	0	12(100.0%)	0	0	12(100.0%)	0	0	0	0	12(100.0%)^2^	0	1(8.3%)	0	0	0	12(100.0%)	0	0
6n	31	0	31(100.0%)	0	0	31(100.0%)	0	0	0	0	31(100.0%)^2^	0	31(100.0%)	0	0	0	31(100.0%)	0	0
6u	7	0	7(100.0%)	0	0	7(100.0%)	0	0	0	0	7(100.0%)^2^	0	7(100.0%)	0	0	0	7(100.0%)	0	0

NI: nucleoside inhibitor; NNI: nonnucleoside inhibitor; NNI-1 and NNI-2 bind to thumb 1 and thumb 2 of NS5B polymerase; NNI-3 and NNI-4 bind to palm 1 and palm 2 of NS5B polymerase; Bold type represents the major substitutions associated with the resistance phenotype (high-intermediate level of resistance).

1, the resistance associated mutation is C316N

2, the resistance associated mutation is I482L

## Discussion

In the present work, we conducted a systematic HCV molecular epidemiological study to reveal the temporal and spatial distribution of HCV subtypes among IDUs co-infected with HIV-1 and HCV in Yunnan Province, China, which is severely affected by HIV/AIDS. The results demonstrate a diversity of HCV genetics in this population and suggest that HCV was multiply introduced through the high-risk behavior of drug injection. Further, we first diclosed that naturally occuring resistance-associated mutations to NS5B polymerase inhibitors existed in the HCV subtypes circulating in China. Our study explored the history of the HCV epidemic in IDUs and could contribute to the prevention and control of HCV in Yunnan Province.

Seven HCV subtypes were found in IDUs co-infected with HIV-1 and HCV. Of these subtypes, 3b and 3a were the most prevalent subtypes. The frequency of 3b was the highest in five prefectures and the second highest in one prefecture. Subtype 3b was found to be the most frequent subtype in this population in the early 2000s [29]. However, it had not been detected in Wenshan Prefecture at that time. In this study, 3b was found in Wenshan, which suggested that its coverage has enlarged. The frequency of subtype 3a was second only to that of subtype 3b. The constituent ratios of subtype 3a ranked first or second in five prefectures, decreasing from the eastern prefectures to the western ones. However, the top two HCV strains in the general population of Yunnan changed from 1b and 3b to 3b and 3a in the 2000s [33], which was similar to the distribution pattern in IDUs.

After 3b and 3a, 1b was one of the top three subtypes in both the general population and in IDUs in the early 2000s [29]. Although the frequency of 1b was found to somewhat decrease in IDUs in this study, 1b is also a main subtype circulating in Yunnan. More importantly, the frequency of 1b is the highest in the whole nation, especially in the general population [11, 33]. These subtypes have developed complicated prevalence patterns because they are also common in the non-IDU population. To better understand the epidemics of these three subtypes in Yunnan, further studies, including phylogeographic analysis, should be performed in the general population.

In total, the frequency of genotype 6 followed that of genotype 3. Besides 3b and 3a, 6n was also found in IDUs of all six prefectures, however, it was seldom reported in the general population [33], which suggests that 6n was a distinctive strain in IDUs in Yunnan. Strikingly, its genetic distance was the largest, and its tMRCA was the earliest, suggesting that 6n was multiply introduced into Yunnan and that it has had the longest circulating times. Phylogeographic analysis of 6n showed that there were at least three independent transmission events, one of which arose in Dehong with a common ancestor dated around the 1910s, and spread from Dehong to the eastern prefectures since 1970s. The other two events initiated in the 1970s; however, they did not widely disseminate: one was limited to Lincang and Dali, the other was limited to Dehong.

Similarly to subtype 6n, subtype 6u and 1a might have originated in Dehong, especially in the Burmese population, which suggested the existence of cross-border transmission. Of the seven subtypes, the circulating time of 6u was the shortest in Yunnan. In fact, as a novel subtype, 6u was first identified and reported in Dehong [29, 34]. In the early 2000s, subtype 1a and 6u were found only among IDUs in Dehong [29]. In this study, the geographic distribution and phylogeographic analyses demostrated that these two subtypes spread from Dehong to the internal area of Yunnan. Furthermore, subtype 6u was also transmitted to the other provinces, such as Henan Province and Jiangsu Province.

A recent study indicated that Vietnam could be the origin of 6a in China [12]. Subtype 6a was first introduced into Guangxi and Yunnan, two provinces bordering Vietnam. From Guangxi, 6a further spread to the neighboring provinces, including Guangdong, Yunnan, Hainan and Hubei. Subsequently, 6a became a local epidemic in Guangdong, which made Guangdong the second source region to disseminate 6a to the other 12 provinces [11, 12]. Because of this, the prevalence of 6a significantly increased in China recently [11, 12, 33]. However, after migrating into Yunnan, 6a was only detected among IDUs in limited regions (Honghe and Wenshan) [29]. In this study, in addition to Honghe and Wenshan, 6a was also detected among IDUs in the central and western prefectures (Kunming and Dehong). The phylogeographic analysis also supports that the isolates detected in the two prefectures were from those in Honghe.

Yunnan is situated along drug trafficking routes that channel heroin into China. It is accepted that China’s first HIV-1 epidemic was initiated among IDUs in Yunnan [27]. Through the drug trafficking routes, the HIV epidemic spread from Yunnan to other parts of China [27]. Some genotypes originally found in Yunnan had spread not just to the neighboring provinces of Guangxi (CRF08_BC) [35–39] and Sichuan (CRF07_BC) [40], but also to the north-western province of Xinjiang (CRF07_BC) [38, 40] and the central province of Henan (subtype B') [41]. Because HCV and HIV shared the same transmission routes, IDUs also drove the rapid transmission of HCV in China [42, 43]. Due to its special geographic location, Yunnan also played an important role in the development of the HCV epidemic in China. According to a nation-wide phylogeographic analysis, 3b showed a trend for migration from the southwest to other regions, which is consistent with the known drug trafficking route in which Yunnan links the Golden Triangle to the inland of China. The existing data show that Yunnan became a second source region to disseminate 3a to the other provinces after 3a was dispersed into Yunnan through the IDUs network [44, 45]. The previous study also indicated that 6n found in eastern China came from Yunnan [44]. This study shows for the first time that 6u found in central China and in eastern China also came from Yunnan.

For both HIV and HCV, Dehong is an area of concern. In the late 1980s and the early 1990s, HIV-1 subtypes B and C were first introduced into Dehong. At that time, intravenous drug injection was the predominant transmission route [27, 46], which provided an opportunity for recombination between subtype B and subtype C among the IDU population. A recent investigation showed that the proportion of unique recombinant forms (URFs) was higher than any other subtype/circulating recombinant form (CRF) in Dehong [47]. Furthermore, some novel CRFs were identified from these URFs, including CRF62_BC, CRF64_BC and CRF65_cpx [48–50]. This study provided evidence that HCV subtype 1a, 6n and 6u among IDUs in Yunnan also initiated from Dehong. Dehong is an earlier opened trading port, where cross-border intercourse is frequent, increasing the possibility of the introduction of infectious diseases. In this study, a certain amount of Burmese staying in Dehong were recruited. Thus, how to improve the prevention and control of HIV and HCV in foreign personnel is an issue of concern.

It is believed that genotype 6 originated in Southeast Asia. In the countries bordering with Yunnan (Myanmar, Laos and Vietnam), genotype 6 is the predominant strain. In addition, genotype 6 showed very high variations in these countries. At least 9 subtypes of HCV genotype 6 (6a, 6d, 6e, 6h, 6k 6l, 6o, 6p and 6t) were reported in Vietnam [51, 52], seven subtypes (6b, 6h, 6i, 6j, 6l, 6o and 6q) in Laos [53], four subtypes (6f, 6m, 6n and 6u) in Myanmar [54, 55]. Thus, it is rational that the subtypes of genotype 6 which detected in Yunnan come from the neighboring countries. However, evolutionary analysis indicates that genotype 3 might have originated in Africa and entered South Asia by the Arabian slave traders around 450 years ago [56]. Subsequently, genotype 3 diverged into several subtypes in India [57]. Presently, genotype 3 is common in the Indian sub-continent (India, Pakistan and Nepal) [58–60]. Historically, HIV-1 subtype C was introduced into Yunnan by Indian IDUs [61, 62], which means that IDUs also could mediate the transmission of HCV genotype 3 from the Indian sub-continent to China. The previous study showed that HCV 3a was initially introduced from India into Xinjiang in 1981.3, and subsequently dispersed into Yunnan [45]. In this study, the tMRCA for 3a in Yunnan was 1973.4, which is earlier than that of 3a in Xinjiang. Thus, it could not be excluded that 3a in Yunnan was directly introduced from the Indian sub-continent.

The HCV genome is comprised of a single open reading frame (ORF), which consists of four structural genes (Core, E1, E2 and p7) and six non-structural genes (NS2, NS3, NS4A, NS4B, NS5A and NS5B). The ORF is first translated to a single polyprotein, which is further processed to into the proteins corresponding to the different genomic regions. HCV is a rapidly-evolving RNA virus. However, the HCV evolutionary rates are different among the various genomic regions. Usually, higher rates were observed in the E2 and P7 regions, while lower rates were seen in the Core and NS5B [63]. In fact, the evolutionary rates in the same genomic region also differed among the various subtypes. As described in this study, the median evolutionary rate of 6u was the highest, and that of 1b was the lowest. This suggests that different subtypes have varied selective pressures. Aside from genetic features, some factors may contribute to the difference, such as immune response, antivirus therapy, or transmission routes.

In theory, HCV is more prone to occur drug resistance than HIV, because the virion production of HCV is 100-fold higher than that of HIV, the error rate of HCV RNA polymerase is approximately 10-fold higher than that of HIV reverse transcriptase [64]. As reported in previous studies [24], NS5B NNIs but not NIs resistance-associated mutations were found as natural polymorphisims in selected subtypes in this study, which suggests that the senstivity to NNIs may be great heterogenous within different subtypes. In fact, the baseline drug resistance mutations to NS3/4A PIs and NS5A inhibitors were also reported in DAAs-naïve patients [25, 26]. These suggest that each genotype/subtype has a specific profile of baseline drug resistance to DAAs, which should be considering in the selection of DAA regimens. If possible, a genotypic drug resistance test should be conducted before starting a DAA regimen. Another issue is that most resistance-associated mutations were identified in genotype 1 [65], the prodominant strain in America and Europe. Because of the viral genetic diversity, the phynotype of these mutations should be verified in different genotypes/subtypes.

In conclusion, this study elucidated the complicated and distinctive HCV genetics and naturally occuring drug resistance among HIV-infected IDUs in Yunnan, and provide important information to develop effective measures for HCV prevention, treatment and care in this population.

## Supporting Information

S1 Table The Bayes Factors between other three coalescent tree priors and constant size coalescent tree prior. (PDF)
