# Supplementary material for: Multiple Introduction and Naturally Occuring Drug Resistance of HCV among HIV-Infected Intravenous Drug Users in Yunnan: An Origin of China’s HIV/HCV Epidemics
Source: PLoS One. 2015 Nov 12;10(11):e0142543. doi: 10.1371/journal.pone.0142543 (PMC4642981; doi:10.1371/journal.pone.0142543)
Supplement: S1 Table — (PDF) [file pone.0142543.s001.pdf]

S1 Table. The Bayes Factors between other three coalescent tree priors and constant size coalescent tree prior.

Coalescent tree priors	ln Bayes Factors						
	1a	1b	3a	3b	6a	6n	6u
Constant Size	-	-	-	-	-	-	-
Exponential Growth	**0.147**	1.716	-8.502	1.031	-0.778	-0.003	1.505
Logistic Growth	-0.485	-0.135	-1.578	-4.437	-0.17	-0.002	2.181
Bayesian Skyline	-1.389	**2.24**	**1.529**	**6.497**	**0.235**	**1.102**	**2.642**
